# Transcriptomic analysis of enteropathy in Zambian children with severe acute malnutrition

**DOI:** 10.1016/j.ebiom.2019.06.015

**Published:** 2019-06-20

**Authors:** Mubanga Chama, Beatrice C. Amadi, Kanta Chandwe, Kanekwa Zyambo, Ellen Besa, Nurmohammad Shaikh, I. Malick Ndao, Philip I. Tarr, Chad Storer, Richard Head, Paul Kelly

**Affiliations:** a Tropical Gastroenterology and Nutrition group, University of Zambia School of Medicine, Nationalist Road, Lusaka, Zambia; b Department of Pediatrics, Washington University School of Medicine, St Louis, MO, United States; c Department of Genetics, Washington University School of Medicine, St Louis, MO, United States; d Blizard Institute, Barts & The London School of Medicine, Queen Mary University of London, 4 Newark Street, London, UK

**Keywords:** Enteropathy, Severe acute malnutrition, Pediatric gastroenterology, RNA sequencing, Solute carriers

## Abstract

**Background:**

Children with severe acute malnutrition (SAM), with or without diarrhoea, often have enteropathy, but there are few molecular data to guide development of new therapies.

We set out to determine whether SAM enteropathy is characterised by specific transcriptional changes which might improve understanding or help identify new treatments.

**Methods:**

We collected intestinal biopsies from children with SAM and persistent diarrhoea. mRNA was extracted from biopsies, sequenced, and subjected to a progressive set of complementary analytical approaches: NOIseq, Gene Set Enrichment Analysis (GSEA), and correlation analysis of phenotypic data with gene expression.

**Findings:**

Transcriptomic profiles were generated for biopsy sets from 27 children of both sexes, under 2 years of age, of whom one-third were HIV-infected. NOIseq analysis, constructed from phenotypic group extremes, revealed 66 differentially expressed genes (DEGs) out of 21,386 mapped to the reference genome. These DEGs include genes for mucins and mucus integrity, antimicrobial defence, nutrient absorption, C-X-C chemokines, proteases and anti-proteases. Phenotype – expression correlation analysis identified 1221 genes related to villus height, including increased cell cycling gene expression in more severe enteropathy. Amino acid transporters and ZIP zinc transporters were specifically increased in severe enteropathy, but transcripts for xenobiotic metabolising enzymes were reduced.

**Interpretation:**

Transcriptomic analysis of this rare collection of intestinal biopsies identified multiple novel elements of pathology, including specific alterations in nutrient transporters. Changes in xenobiotic metabolism in the gut may alter drug disposition. Both NOIseq and GSEA identified gene clusters similar to those differentially expressed in pediatric Crohn's disease but to a much lesser degree than those identified in coeliac disease.

**Fund:**

Bill & Melinda Gates Foundation OPP1066118. The funding agency had no role in study design, data collection, data analysis, interpretation, or writing of the report.

**Research in context**

**Evidence before this study**

Children in Africa and Asia with severe acute malnutrition (SAM) complicated by overt infectious disease (diarrhoea, measles, pneumonia) have continuing unacceptably high mortality rates. Several lines of evidence implicate intestinal dysfunction in driving mortality. A systematic search revealed very few studies which have analysed the small intestinal mucosa to identify pathophysiological processes which could contribute to adverse outcomes.

**Added value of this study**

In this study of 27 children with SAM and persistent diarrhoea, RNA sequencing was used to correlate transcriptomic profiles with phenotypic measures of intestinal dysfunction, including villus height, circulating lipopolysaccharide concentrations, lactulose:rhamnose ratio, and circulating IgG antibodies to deamidated gliadin peptides. Differentially expressed genes and gene sets included amino acid and zinc transporters, mucosal protection molecules, cell proliferation genes, chemokines, and xenobiotic metabolising enzymes.

**Implications of all the available evidence**

While previous studies of intestinal dysfunction in severe malnutrition have drawn attention to mucosal abnormalities, including increased permeability, unsupervised transcriptomic analysis has revealed specific alterations in physiological processes which could inform the search for improved therapy for this life-threatening disorder.

Alt-text: Unlabelled Box

## Abbreviations

**Table:** 

SAM	severe acute malnutrition
DEGs	differentially expressed genes
GSEA	gene set enrichment analysis
LPS	lipopolysaccharide
LBP	LPS binding protein
FABP	intestinal fatty acid binding protein
VH	villus height
LR	lactulose:rhamnose recovery ratio
DGP	deamidated gliadin peptides

## 1 Introduction

Malnutrition in children in low- and middle-income countries persists on a huge scale [1]. Stunting, impaired linear growth, affects 159 million children, 24% of all the world's children [1]. Wasting is less common, but severe acute malnutrition (SAM) represents a medical emergency. Optimal treatment of SAM in the community is associated with mortality rates around 4% per episode but once medical complications supervene in-hospital mortality climbs to 10%–40% [[2], [3], [4]]. Children with SAM and persistent diarrhoea have particularly high mortality rates, as do children with SAM and HIV^5^. Counter-intuitively, malnutrition does not fully respond to nutritional therapy. Stunting rates can only be reduced by 15% at most even with optimally-delivered nutritional care [6] or sanitation [7,8]. Wasting (SAM) is associated with high relapse rates following discharge [9]. There are three major possible explanations for why improved nutrition is insufficient to treat malnutrition. First, insufficient care after discharge from SAM treatment may result in inadequate supplementary feeding. Community-based management of acute malnutrition should overcome this [10,11]. Second, children return to the same environment from which they came so economic, parenting, and environmental factors continue to play an adverse role. Third, there may be some specific biological obstacle to recovery which renders nutritional therapy ineffective, or only partially effective. We recently demonstrated that in SAM there is severe small intestinal enteropathy, characterised by villus blunting, leakiness, microbial translocation, and mucosal and systemic inflammation [12]. Evidence from Malawi also indicates that intestinal damage plays a central role in mortality in SAM [3].

Older studies indicated that enteropathy is severe in children with SAM [13,14], but few studies have been informed by tissue investigation due to the considerable difficulties in obtaining biopsy material ethically. Here we report the results of a transcriptomic analysis of the same children whose biopsies we characterised in a previous report [12]. The mRNA profile of these biopsies shows consistent patterns across several domains of pathology, highlighting several important cross-cutting themes related to mucus layer integrity, antimicrobial defence, nutrient transporters, protease/anti-protease balance, xenobiotic metabolism, and immune activation, among others. Because samples such as these are difficult to obtain, and we do not consider it justifiable to collect biopsies from healthy children, the analysis which follows compares more severe enteropathy against less severe.

## 2 Methods

Children were recruited from the malnutrition ward of the University Teaching Hospital, Lusaka. This ward treats children with severe acute malnutrition with medical complications, while uncomplicated SAM is treated in the community [11]. Consequently, these children have severe disease and mortality rates have historically been high [15], in common with many other similar centres in Africa [3,5,16]. Up to one half of the children on this ward have HIV infection at any one time [15], which further increases risk of mortality [5,15]. Children were recruited on the basis that they had SAM complicated by persistent diarrhoea, and that initial investigations had revealed nothing to explain their clinical state. Parents/guardians of all eligible children were invited to participate, and <10% declined. Written, informed consent was obtained from parents and caregivers in all cases. Ethics approval was obtained from the University Biomedical Research Ethics Committee on 11th April 2013, reference number 006–01-13.

### 2.1 Investigations

Children with SAM, with or without HIV and with or without oedema, whose parents or guardians had given written consent, underwent endoscopy under sedation with a Pentax EG2490k gastroscope (external diameter 8 mm). Biopsies were collected from the distal duodenum as previously reported [12]. Prior to withdrawal of the endoscope, a test solution containing 1 g lactulose and 0.2 g rhamnose in 20 ml was flushed down the working channel into the duodenum. Blood collected on the day of endoscopy was used for measurement of lipopolysaccharide (LPS), LPS binding protein (LBP), intestinal fatty acid binding protein (FABP), soluble CD14 (sCD14) and CD163 as previously described [12]. Urine was collected after 60–120 min for lactulose:rhamnose (LR) ratio analysis using mass spectrometry [17], as a measure of permeability.

### 2.2 Assessment of inter-biopsy variability

In 13 pairs of biopsies the individual biopsies could be retrieved from the cryo-storage vials and were individually sequenced. These were analysed separately to assess inter-biopsy variation, but then mRNAs were pooled in silico particularly for the analysis of differential expression.

### 2.3 RNA sequencing

Two small intestinal biopsies were immediately snap-frozen in liquid nitrogen prior to storage at -80 °C. RNA was extracted using Trizol reagents (Invitrogen, Carlsbad, CA) followed by silica column purification (RNeasy Mini Kit, Qiagen) and quantified using a Nanodrop spectrophotometer (ND-2000c, Thermo Scientific, USA), prior to transport to the Beijing Genomics Institute (BGI). RNA quality control was performed using an Agilent 2100 Bioanalyser and ABI StepOnePlus Real-Time PCR System. For RNA sequencing preparation, the total RNA was treated with DNase I followed by mRNA enrichment using oligo-dT labelled beads and ligation of sequencing adaptors to the enriched mRNA fragments. RNA sequencing was carried out using an Illumina HiSeq2000 platform with 50 bp reads. Filtering steps included removing reads with adapters, removing reads in which unknown bases were >10%, and removing low quality reads (percentage of low quality bases over 50%). The proportion of clean reads was never <99%, usually >99.6%. After low-quality reads were removed and adapter sequences trimmed 3.3 billion clean reads were obtained, with an average of 165 million reads per sample.

### 2.4 Principal component analysis

Principal Component Analysis (PCA) (Partek Software) was carried out on the FPKM values of all genes with a median value >1.0 across all samples to establish the similarity of replicate samples collected from a single subject.

### 2.5 Definition of group extremes for categorical analysis

It was not possible to identify a group of children to act as controls from whom biopsies could ethically be collected, thus extremes of the ranges were compared across several measurements (i.e severe vs mild enteropathy). Measurement of villus height, LR ratio, LPS and circulating antibodies to deamidated gliadin peptides (anti-DGP) were as previously reported [12]. For each variable, the study cohort was divided into quartiles, with approximately the same number in each quartile but depending on the distribution of values which were sometimes close together. The highest and lowest quartiles were used as the extreme groups. A group with short villi (n = 7) was defined as <180 μm, and a group with long villi (n = 8) defined as >230 μm. A group with high LR ratio (n = 6) was defined as >0.25, and a group with low LR ratio (n = 6) defined as <0.2. A group with high plasma LPS (n = 8) was defined as >300 EU/ml, and a group with low LPS (n = 6) as <120 EU/ml. A group with higher anti-DGP (n = 7) was defined as >8 U/ml, and a group with low anti-DGP (n = 8) was defined as <1.5 U/ml.

### 2.6 Transcriptomic analysis - gene expression and differential expression

Gene expression levels and differentially expressed genes (DEGs) were identified in an independent analysis at the Beijing Genomics Institute. Expression levels were quantified by Expectation Maximization (RSEM) software and expressed in Fragment Per Kilobase of transcript per Million (FPKM). Differentially expressed genes (DEGs) were identified between group extremes, defined as above, using NOIseq [18] analysis. Using log_2_-fold change, a heatmap was then constructed from these groups, including only those genes with probability of difference of 0.8 or more.

### 2.7 Phenotypic correlation analysis

The enteropathy phenotypes assessed included average villus height, LR ratio, circulating lipopolysaccharide levels, and circulating IgG anti-DGP levels. Additionally, age, history of breastfeeding, and HIV status were examined. Enteropathy phenotypes were correlated against the RNAseq gene expression data using Spearman's rank correlation and were filtered for significance using a correlation p-value of p < 10^−6^ and a Spearman's correlation coefficient (ρ) ≥0.4 or ≤−0.4. Genes with a maximum FPKM <10.0 were not considered for further functional analysis.

### 2.8 Biological process and pathway analysis

DEGs were manually grouped according to known function in the small intestine using KEGG, Reactome and GeneCards databases. In addition, networkanalyst™/DAVID was employed to annotate DEGs according to KEGG and Reactome. Results were plotted in R version 3.3.1. Protein-protein interactions networks (first-order) among the DEGs were constructed with networkanalyst™ using the InnateDB with a confidence score cutoff of 900. The nodes and edges generated were then edited in Gephi version 0.91. Furthermore, functional class scoring was carried out using Gene Set Enrichment Analysis (GSEA v3.0 Beta 2) software from the Broad Institute. Gene expression data between groups and genes from the phenotype correlation analyses were compared against Hallmark gene sets of Molecular Signature Database (MSigDB) using signal to noise metric ranking for gene sets and permuted 100 times by phenotype class.

### 2.9 Data sharing

Annotated datasets are available on request from the corresponding author.

## 3 Results

Transcriptomic profiling was carried out on biopsy sets from 27 children with SAM of both sexes, under 2 years of age, of whom one-third were HIV-infected (Table 1).

**Table 1:** Clinical and demographic characteristics of children with SAM.

Characteristic	By sex			By HIV serological status		
	Male *n* = 16	Female *n* = 11	*P*	HIV seronegative *n* = 18	HIV seropositive *n* = 9	*P*
Sex (M:F)	–	–	–	11:7	5:4	1.00
Age (months)	14 (10,19)	19 (12,21)	0.21	15 (11,20)	12 (10,21)	0.88
HIV seropositive	5	4	1.00	–	–	–
Current breastfeeding	3	0	0.25	1	2	1.00
WAZ	−3.4 (−5.1,−2.5)	−3.6 (−4.7,−2.7)	0.98	−3.2 (−4.0,−2.7)	−4.6 (−5.0,−3.3)	0.16
LAZ	−2.5 (−3.8,−2.0)	−3.0 (−4.4,−1.6)	0.92	−2.5 (−3.7,−1.7)	−3.8 (−5.5,−2.3)	0.22
WLZ	−2.8 (−4.0,−1.7)	−2.8 (−4.2,−1.0)	0.92	−2.7 (−3.5,−1.4)	−2.8 (−4.4,−2.5)	0.35
VH (μm)	216 (143,247)	215 (164,246)	0.96	202 (153,247)	225 (175,243)	0.85
LPS (EU/ml)	339 (57,556)	183 (120,267)	0.45	272 (142,600)	120 (0.223)	0.06
L:R ratio	0.289 (0.170, 1.000)	0.189 (0.104, 0.401)	0.23	0.213 (0.129, 0.289)	0.762 (0.357, 1.000)	0.23
Anti-DGP IgG	2.5 (1.5, 8.6)	2.9 (1.4, 20.7)	0.89	1.6 (1.6, 3.6)	26.0 (5.1, 45.7)	0.005
Lactase deficiency	5/12 (42%)	2/5 (40%)	1.00	6/13 (46%)	1/4 (25%)	0.60

Continuous variables are shown as median (interquartile range). LPS, lipopolysaccharide; VH, villus height.

### 3.1 Characterization of mild and severe domain groups

Characteristics of the groups constructed at the extremes of the selected domains (villus height, circulating endotoxin, permeability, and anti-deamidated gliadin peptide) are shown in Supplementary Table S1. These groups demonstrated very little overlap (Supplementary Fig. S1). For example, groups widely separated by villus height do not differ appreciably in terms of systemic inflammatory or bacterial translocation markers (Table S1).

### 3.2 Principal component analysis (PCA) of repeat and singleton biopsies, and HIV

**Fig. 1:**
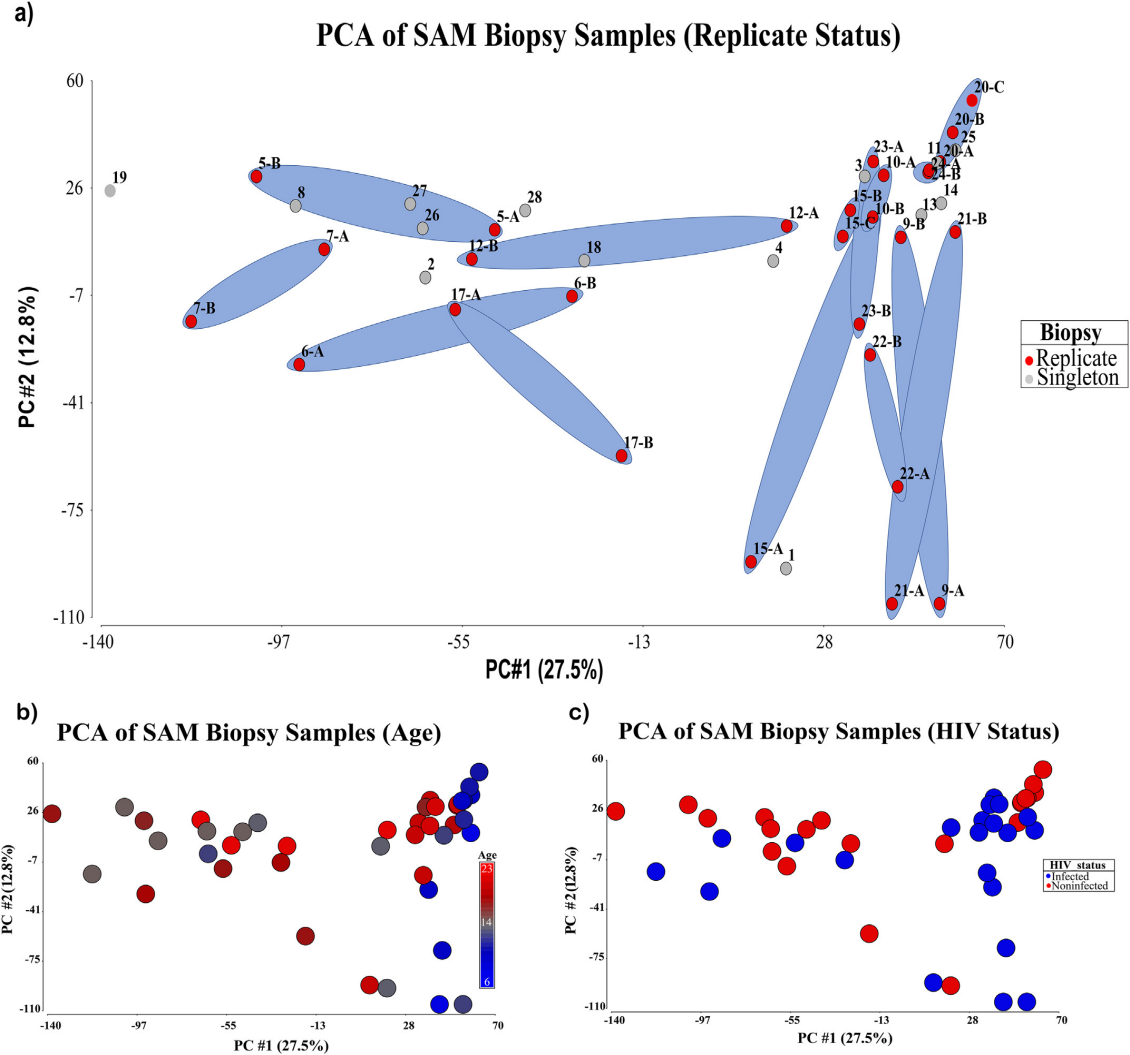
Principal Component Analysis of transcriptomes of Zambian children with Severe Acute Malnutrition. (A) pairs of duplicate biopsies from the same child, taken on the same day, are ringed; only one pair shows a difference in both components between biopsies from the same child. (B) the influence of age on transcriptomes. (C) the influence of HIV on the transcriptome, showing no discernible pattern.

PCA analysis was performed on all samples, repeat and singleton, utilizing all genes with a median FPKM ≥1.0 across the sample. A total of 40.3% of the variation was described in the first two components of the analysis and as can be seen in Fig. 1A. While several of the biopsies exhibited a degree of variation in either the first or the second component, only subject p17 appeared to have substantial variation across both of the first two components. Viewing demographic data overlaid onto the map suggested that age differences were roughly associated with approximate grouping of subject samples across component 1 (Fig. 1B), and HIV had minimal influence on the observed variation (Fig. 1C).

### 3.3 NOISeq identification of DEGs in enteropathy within domain groups

**Fig. 2:**
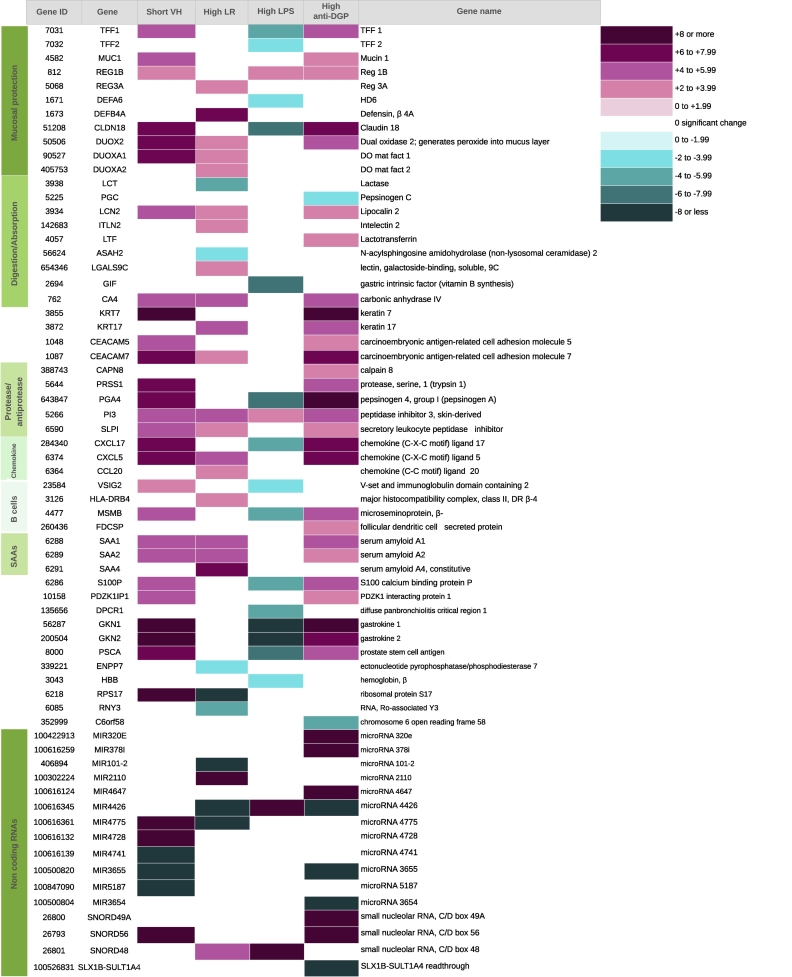
Differentially expressed genes identified by NOIseq analysis. Heat map of 66 differentially expressed. Colours represent the log2 –fold change in more severe enteropathy (short villi, high lactulose permeation, high plasma lipopolysaccharide, and higher blood anti-deamidated gliadin peptide concentration).

NOIseq analysis identified 66 significantly differentially expressed genes (DEGs), of 21,386 mapped to the reference genome, using a probability of difference cut-off of 0.8 or more (Fig. 2). The manual functional classification of these DEGs (Fig. 2) reveals gene groups that are implicated in mucus biology, antimicrobial defence, protease/anti-protease balance, CXC chemokine secretion, B cell function, and serum amyloid A. Further analysis of some of these functional groups is described below. Of note, the pattern of changes differed for LPS to that seen in the other domains (VH, LR ratio, anti-DGP). It is also striking that the magnitude of the change was greater for non-coding transcripts. Further analysis with Network analyst revealed predicted interactions of these DEGs with each other and with other genes with they are closely associated in the literature (Supplementary Figs. S7-S10). NOIseq analysis comparing HIV-infected and uninfected children with SAM found 41 differentially expressed genes (Supplementary Table S2).

### 3.4 Phenotypic correlation analysis

**Fig. 3:**
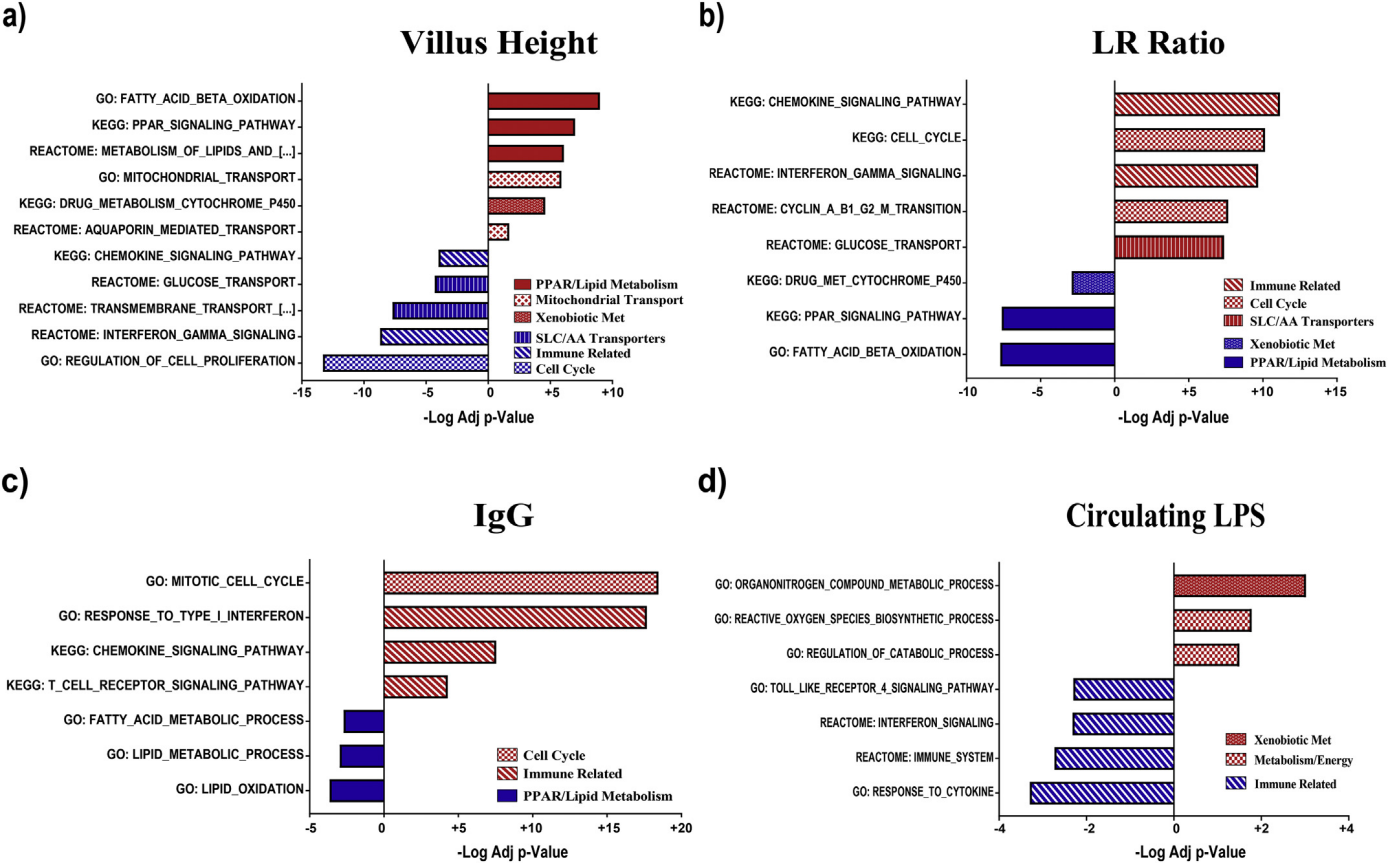
Pathway correlations with measures of enteropathy: (A) villus height, (B) permeability measured as lactulose:rhamnose (LR) ratio, (C) anti-deamidated gliadin peptide IgG, and (D) circulating LPS as a marker of translocation.

As described in the methods a correlative analysis was performed for each of the domains, without grouping, to account for the continuous nature of the data. A non-parametric Spearman analysis identified 1221 genes correlated with VH; 3043 correlated with LR ratio (permeability); and 1491 associated with anti-DGP IgG with p < 10^−6^. Correlation with circulating LPS demonstrated fewer significant genes (269), nearly an order of magnitude less. Given the much larger number of highly correlated genes with the phenotypic domains as compared to DEGs identified via predefined groups based on fixed cutoffs, GSEA analysis was employed to provide a comprehensive overview of the key processes correlated either positively or negatively with the subject values (Fig. 3). Again, highly significant processes and pathways associated with multiple domains included cell-cycle/proliferation, immune cell activity, absorptive mechanisms, mucosal barrier, and xenobiotic metabolism.

### 3.5 Cell-cycle and proliferation associated genes

Among the list of key processes and pathways identified by GSEA as correlated with LR ratio and inversely with VH are multiple entries associated with cell cycle and proliferation (Fig. 3). The number and directionality of these genes, which included proliferating cell nuclear antigen, cyclins, cyclin-dependent kinases and p53 related transcripts (Supplementary Fig. S2) suggest that malnutrition enteropathy is a hyper-proliferative (hyperplastic) rather than atrophic enteropathy.

### 3.6 Dysregulation of immune responses

In view of past studies suggesting that environmental enteropathy is related to T cell activation (and hyperproliferation) [[19], [20], [21]], and NOIseq data implicating immune pathways (Figs. 2 and 3), we interrogated GSEA results to identify immunological genes demonstrating correlation with VH, LR, LPS, and anti-DGP IgG. A range of genes were identified that represent a broad range of immune responses, including chemokines, cytokines, antigen presentation and immune cell markers (Supplementary Fig. S3). However, few significant correlations were observed with LPS.

### 3.7 Dysregulation of absorptive mechanisms

Given that the principal function of the intestine is nutrient absorption, we analysed transcripts for known solute carriers (SLC genes) to search for correlations with VH or LR. The majority of mitochondrial transporters (SLC family 25 family) were positively correlated with VH and negatively with permeability and therefore consistent with expected changes in enterocyte mass. In contrast, a broad range of amino acid transporters were inversely correlated with VH and positively with permeability, suggesting specific up-regulation (Supplementary Fig. S4). The ZIP family of zinc transporters, with the exception of ZIP-4, followed the pattern of the amino acid transporters, as did one choline and one riboflavin transporter (Fig. S4). However, the bidirectional zinc transporter ZnT-5, which is expressed at the apical membrane, exhibited the pattern consistent with enterocyte mass (i.e. positively correlated with VH).

### 3.8 Mucosal barrier disruption

To explore further the prior hypothesis that mucus barrier disruption is a feature of severe enteropathy (reference 21, Fig. 2 and Supplementary Figs. S7 and S10) we examined mucus constituents, goblet cell transcription factors, and host defence peptides. The majority of these transcripts demonstrated increased expression in more severe enteropathy: inverse correlation with VH and direct correlation with permeability (Supplementary Fig. S5). As with the NOIseq analysis, the pattern of correlation with circulating LPS was not entirely consistent with the pattern of correlation with other domains of pathology. Tight junction and gap junction molecules were distinctly under-represented in the significant correlations identified (Fig. S5). Occludin, ZO-1, E-cadherin and β-catenin were not represented in the database of correlated gene transcripts.

### 3.9 Globally reduced expression of genes for xenobiotic metabolism in severe enteropathy

Genes encoding enzymes required for xenobiotic metabolism demonstrated broad, consistent direct correlations with VH and inverse correlations with LR (Fig. 2; Supplementary Fig. S6), consistent with reduced expression in severe enteropathy.

## 4 Discussion

We carried out a transcriptomic analysis of small intestinal samples taken from Zambian children being treated for SAM in the University Teaching Hospital, Lusaka, Zambia. We undertook this work in the hope that we would gain new insights into pathophysiology, and that we would identify new therapeutic targets. The first analysis of our data using NOIseq identified 66 differentially expressed genes which fall into several functional groups, including nutrient transporters, enzymes for xenobiotic metabolism, and genes with important roles in the integrity of the mucus barrier. A correlative approach identified an order of magnitude more transcripts and a much broader range of implicated gene products. For some gene sets there was a surprisingly high degree of consistency in correlations with VH and LR ratio. Our data are consistent with previous evidence that environmental/malnutrition enteropathy in Africa is a hyperplastic enteropathy characterised, perhaps driven, by immune activation [[19], [20], [21]]. However, additional evidence beyond mRNA analysis is required to establish that this is really the case in children with SAM. If this is correct, we can only speculate on how malnutrition is sensed in the gut, whether by mTOR or by intestinal cell kinase [22]. A recent faecal transcriptomic study from Malawi also implicated immune activation (HLADR, CD53, TNF) and mucosal protection (MUC12, S100A8 and Reg1A) [21]. The changes in immune genes are not those which would be expected to be associated with major infiltrations of neutrophils or eosinophils, and were not strongly related to LPS, our biomarker of microbial translocation.

It is of considerable interest that several sets of genes showed an apparently specific up-regulation, such as that noted for amino acid and zinc transporters. Some transporters, such as mitochondrial transporters, were positively correlated with VH, which we believe signifies that expression levels of these enzymes reflect enterocyte mass, not necessarily gene expression change. The inverse correlations observed for plasma membrane amino acid transporters suggests specific differential regulation, and that enterocytes have responded to greater amino acid demand, or reduced availability, or both. We interpret the general increase in ZIP proteins in the same way, though the functions of different ZIP proteins are complex, and ZIP-4 has been shown to be up-regulated in zinc deficiency [23]. Its correlation here is of interest because these children were all being given supplementary zinc as part of the nutritional rehabilitation they were undergoing. ZnT-5 is potentially a bidirectional transporter located at the enterocyte luminal surface [23], so the significance of its negative correlation with permeability remains uncertain, as does the physiological impact of altered zinc transporter expression. Of course, as this is an analysis of mRNA only, increased gene expression may signify either up-regulation as part of pathophysiology or a compensatory response to increased protein degradation or shedding. Further work is needed to clarify the physiological consequences of the transcriptional changes we have identified.

There was considerable overlap between the NOIseq transcriptomic profile in SAM (Fig. 2) and that previously reported by RNA sequencing in children with IBD [24]. In SAM enteropathy and in Crohn's disease there was increased expression of DUOX2, DUOXA2, lipocalin 2, CEACAM 5 and 7, MUC1, SAA 1, 2 and 4, and CXCL5 genes (Supplementary Figs. S2–5, S7 and S10), and these transcripts were further over-expressed in HIV infection (Supplementary Table S2). We conducted a systematic search for transcriptomic studies in IBD and found 3 studies in children and four in adults, mostly by microarray (Supplementary table S3). DUOX, MUC, S100 and Reg families were significantly increased in IBD in four of these studies (Table S3). MUC1 is a membrane-bound mucin [25,26] with widespread expression, including small intestine. DUOX2 is required for synthesis of thyroid hormone, but it also generates reactive oxygen species which play a role in mucosal defence [27]. It is induced by TNFα and MDP and protects against infection with *Listeria monocytogenes* [28]. It also mediates the induction of mucins by epidermal growth factor (EGF) [29]. Reactive oxygen species synthesised by NADPH oxidases (including DUOX2) reduce virulence in *Campylobacter jejuni* [30]. Serum amyloid A (SAA) proteins include three isoforms: 1, 2 and 4 (SAA3 is a pseudogene) and they act as acute phase reactants [31] in Crohn's disease and many other inflammatory disorders. Despite the morphological similarity between severe EE and early coeliac disease, their transcriptomic profiles demonstrate limited overlap. In an analysis of peripheral blood mononuclear cells in coeliac disease, interferon-γ and BACH2 were identified as central to the gene expression changes [32]. In another study, DEGs included CD19, CD203c, HLAE, DEFA5, IFNG, and IL18 [33]. In contrast, these were not dominant DEGs in SAM enteropathy.

Another readily identifiable functional cluster relates to antimicrobial protection of the mucosa. DEFA6 encodes human defensin 6, a Paneth cell product with antimicrobial properties due to its ability to enmesh bacteria [34]. DEFB4A encodes human β-defensin 2, an antimicrobial peptide expressed at mucosal surfaces which is inducible during inflammation but which we have previously reported to be expressed only at low levels in environmental enteropathy [35]. Reg 1A & B and Reg 3A are related antimicrobial peptides with predominant distribution in the pancreas and small intestine (Paneth cells) respectively [36,37]. Lactoferrin possesses antibacterial properties through sequestration of Fe, an important bacterial nutrient, and through interruption of dsRNA genomes [38]. MSMB is fungicidal.

Principal components analysis shows that, although there is variation in gene expression profiles between biopsies taken from the same child on the same day, paired biopsies from one child are much closer together than biopsies from different children. This may be of importance for future work. Although it seems that one single biopsy does represent the transcriptional profile of that individual's mucosa at that point in time, there was considerable variability and it would be desirable to include more than one biopsy in any transcriptomic experiment.

HIV was not the focus of this study, but HIV infected children are at much higher risk of complicated SAM and therefore are over-represented in a group of children such as this. The transcriptional changes attributable to HIV suggest that in addition to the higher expression of certain genes (including NADPH oxidases) there is even higher expression in HIV infection. We know that there is a functionally important mucosal immune defect in these children as their response to treatment for cryptosporidiosis is dramatically impaired [39]. However, the nature of that defect is not clear [40]. Our data suggest that HIV induces additional inflammation on top of that induced by SAM, and alters protease secretion. There are also clear signals from genes which are not well understood, including gastrokines.

Our findings, though based on a small number of children, may suggest novel therapeutic approaches to encourage mucosal healing in SAM. Enhancement of mucus integrity and antimicrobial defence may be worthwhile avenues for future research. Immune activation has long been recognised as a mediator of enteropathy [19,20], and suppression of T cell activation using a mucosal immunosuppressant needs to be evaluated in a clinical trial. Further analysis of our data, available from the authors, may reveal additional novel targets for development of therapies.

## Appendix A Supplementary data

Supplementary material Image 1
