# Supplementary material for: Transcriptomic analysis of enteropathy in Zambian children with severe acute malnutrition
Source: eBioMedicine. 2019 Jun 20;45:456–63. doi: 10.1016/j.ebiom.2019.06.015 (PMC6642221; doi:10.1016/j.ebiom.2019.06.015)
Supplement: Supplementary file 1 — Supplementary material [file mmc1.docx]

**Transcriptomic analysis of enteropathy in Zambian children with severe acute malnutrition**

**Supplementary material**

**Table S1 Characteristics of groups defined as extremes in each domain for NOIseq analysis**

|  | VH | LR | LPS | anti-DGP |
| --- | --- | --- | --- | --- |
| **Severe enteropathy** | | | | |
|  | Short villi | High LR | High LPS | High anti-DGP |
| n | 7 | 6 | 8 | 7 |
| Sex (M:F) | 5:2 | 5:1 | 7:1 | 5:2 |
| Age (months) | 9 (6-23) | 13.5 (9-21) | 12.5 (9-16.5) | 12 (9-15) |
| WLZ | -2.8 (-4.2,-2.3) | -2.8 (-3.9,-1.8) | -3.0 (-4.2,-1.2) | -2.8 (-4.4,-1.8) |
| LAZ | -3.1 (-3.7,-2.7) | -2.7 (-3.8,-2.3) | -2.4 (-3.4,-2.0) | -2.8 (-4.4,-2.3) |
| HIV infected | 2 | 3 | 1 | 4 |
| VH (μm) | 143 (83-165) | 83 (74-165) | 230 (143-246) | 83 (74-215) |
| L:R ratio | 0.630 (0.184-1.01) | 0.762 (0.306-1.05) | 0.213 (0.147-0.272) | 0.954 (0.272-1.05) |
| LPS (EU/ml) | 257 (57-339) | 237 (57-339) | 578 (399-1136) | 130 (57-556) |
| anti-DGP | 3.6 (2.4-8.6) | 10.5 (5.1-15.7) | 3.3 (1.3-10.4) | 15.6 (8.6-45.7) |
| CRP (μg/ml) | 0.8 (0.5-1.5) | 0.8 (0.5-6.0) | 1.2 (0.7-3.3) | 0.1 (0.05-0.6) |
| sCD14 (μg/ml) | 2.5 (1.5-3.2) | 1.6 (1.1-1.8) | 2.6 (2.0-3.3) | 2.2 (1.8-3.2) |
| CD163 (μg/ml) | 1.7 (1.1-2.0) | 1.2 (0.6-1.8) | 1.2 (0.9-1.4) | 0.9 (0.6-1.4) |
| FABP (ng/ml) | 2.8 (1.6-3.4) | 2.9 (2.7-3.4) | 2.8 (2.1-3.9) | 3.4 (2.7-4.3) |
| **Mild enteropathy** | | | | |
|  | Tall villi | Low LR | Low LPS | Low anti-DGP |
| n | 8 | 6 | 6 | 8 |
| Sex (M:F) | 5:3 | 3:3 | 4:2 | 5:3 |
| Age (months) | 12 (9.5-16.5) | 18 (11-21) | 19.5 (12-21) | 16 (12-21) |
| WLZ | -1.3 (-3.7,-0.5) | -4.0 (-5.0,-2.3) | -3.7 (-4.5,-2.8) | -2.7 (-5.2,-0.8) |
| LAZ | -2.7 (-4.3,-1.6) | -3.3 (-3.7,-2.3) | -3.0 (-5.5,-1.9) | -2.1 (-3.8,-1.6) |
| HIV infected | 3 | 1 | 3 | 0 |
| VH (μm) | 247 (244-268) | 230 (215-246) | 175 (74-215) | 246 (244-247) |
| L:R ratio | 0.147 (0.111-0.232) | 0.146 (0.111-0.193) | 1.05 (0.148-1.07) | 0.147 (0.111-0.233) |
| LPS (EU/ml) | 650 (460-1622) | 555 (263-1622) | 0 (0-57) | 272 (142-650) |
| anti-DGP | 1.2 (1.1-1.3) | 2.1 (1.1-3.2) | 5.6 (2.4-15.7) | 1.4 (1.2-1.5) |
| CRP (μg/ml) | 10.0 (1.3-19.5) | 2.8 (1.0-4.9) | 0.6 (0.5-2.6) | 0.3 (0.1-0.7) |
| sCD14 (μg/ml) | 2.6 (2.4-3.2) | 3.0 (2.9-3.4) | 2.4 (1.8-3.4) | 2.6 (2.4-3.5) |
| CD163 (μg/ml) | 1.0 (0.8-1.2) | 1.1 (1.1-1.2) | 1.1 (0.9-1.5) | 1.2 (1.0-2.5) |
| FABP (ng/ml) | 3.4 (3.1-4.3) | 3.2 (2.6-5.1) | 4.4 (3.4-5.1) | 3.4 (2.7-4.9) |

Significance tests were not performed across groups as these groups were not assembled so as to be mutually exclusive (some adults are represented in more than one group), and for some variables the groups were assembled using these measurements as criteria.

**Figure S1 Minimal overlap between phenotypic groups constructed using extremes**

Domains of measurement showing extent of overlap: (A) overlap of most severe groups in each of five domains of measurement; (B) overlap of least severe groups in each of five domains of measurement.

**Table S2 Genes differentially expressed in children with SAM with or without HIV infection**

| **Symbol** | **GeneID** | **Description** | **Mean FPKM HIV positive** | **Mean FPKM HIV negative** | **log2ratio (HIVpos/neg)^*^** | **Probability^§^** |
| --- | --- | --- | --- | --- | --- | --- |
| PSCA | 8000 | Prostate stem cell antigen | 311.408966 | 6.75574429 | 5.526552 | 0.98425043 |
| GKN1 | 56287 | Gastrokine 1 | 91.4971217 | 0.09611564 | 9.8947395 | 0.96282768 |
| IFI6 | 2537 | Interferon, alpha-inducible protein 6 | 1740.44661 | 430.802464 | 2.0143592 | 0.82126469 |
| PI3 | 5266 | Peptide inhibitor 3, skin-derived | 341.86278 | 29.2770863 | 3.5455735 | 0.94818145 |
| TFF1 | 7031 | Trefoil factor 1 | 917.094433 | 93.2593237 | 3.2977504 | 0.94189242 |
| SAA1 | 6288 | Serum amyloid A1 | 607.116756 | 61.9804318 | 3.2920893 | 0.93939309 |
| ISG15 | 9636 | ISG15 ubiquitin-like modifier | 4685.89942 | 907.864363 | 2.3677773 | 0.86940154 |
| DUOXA2 | 405753 | Dual oxidase maturation factor 2 | 266.712304 | 26.3119848 | 3.3414924 | 0.93555488 |
| S100P | 6286 | S100 Calcium binding protein P | 590.661467 | 66.9361474 | 3.1414741 | 0.93038719 |
| GKN2 | 200504 | Gastrokine 2 | 48.1985668 | 0.55529822 | 6.4395837 | 0.92924237 |
| MT1B | 4490 | Metallothionein 1B | 99.4742968 | 733.660367 | -2.88271665 | 0.91372756 |
| DUOXA1 | 90527 | Dual oxidase maturation factor 1 | 81.5243009 | 8.05774909 | 3.3387814 | 0.9079143 |
| SAA2 | 6289 | Serum amyloid A2 | 211.782274 | 27.6793284 | 2.935701 | 0.90776954 |
| CLDN18 | 51208 | Claudin 18 | 40.0945592 | 2.06138416 | 4.2817212 | 0.89957901 |
| MSMB | 4477 | Microseminoprotein beta | 54.0599003 | 4.69534761 | 3.525255 | 0.89689735 |
| MIR4728 | 100616132 | MicroRNA 4728 | 0.07373983 | 31.0920919 | -8.71988783 | 0.89671346 |
| DUOX2 | 50506 | Dual oxidase 2 | 140.679378 | 20.5860274 | 2.7726735 | 0.8871598 |
| SLPI | 6590 | Secretory leukocyte peptidase inhibitor | 111.686842 | 17.1301436 | 2.7048501 | 0.87555441 |
| CXCL17 | 284340 | Chemokine (C-X-C motif) ligand 17 | 25.7061972 | 0.85220337 | 4.9147746 | 0.87075763 |
| PGA4 | 643847 | Pepsinogen 4, group 1 (pepsinogen A) | 22.8151042 | 0.02238309 | 9.9933641 | 0.86614395 |
| PRSS1 | 5644 | Protease, serine 1 (trypsin 1) | 32.0399573 | 2.50921062 | 3.6745667 | 0.86528084 |
| CA4 | 762 | Carbonic anhydrase IV | 29.2729929 | 1.98715788 | 3.8807919 | 0.86402179 |
| MIR4451 | 100616349 | MicroRNA 4451 | 22.3800392 | 0.04937447 | 8.8242316 | 0.86390676 |
| MIR3655 | 100500820 | MicroRNA 3655 | 0.07373983 | 22.2130808 | -8.23474964 | 0.86295757 |
| LCN2 | 3934 | Lipocalin 2 | 1575.02703 | 318.149177 | 2.3076013 | 0.86123918 |
| CEACAM7 | 1087 | Carcinoembryonic antigen-related cell adhesion molecule 7 | 41.0349878 | 4.63527534 | 3.1461276 | 0.86022035 |
| OASL | 8638 | 2’-5’-oligoadenylate synthetase-like | 102.631591 | 17.5034146 | 2.5517666 | 0.85931186 |
| KRT17 | 3872 | Keratin 17 | 23.7334109 | 1.16013549 | 4.3545543 | 0.85481243 |
| MUC1 | 4582 | Mucin 1 | 304.836044 | 65.9473414 | 2.2086471 | 0.84083838 |
| SNORD48 | 26801 | Small nuclear RNA, C/D Box 48 | 0.07373983 | 16.8682943 | -7.83765424 | 0.82985586 |
| LTF | 4057 | Lactotransferrin | 52.8203337 | 9.13394797 | 2.5317829 | 0.82970171 |
| MX2 | 4600 | H6 Family homebox 2 | 101.371131 | 21.3939583 | 2.2443715 | 0.8252774 |
| C6orf58 | 352999 | Chromosome 6 open reading frame 58 | 86.9938301 | 367.314958 | -2.07803266 | 0.82466391 |
| LGALS9C | 654346 | Lectin, galactoside-binding, soluble 9C | 168.424236 | 38.3845367 | 2.1335026 | 0.82240872 |
| CXCL11 | 6373 | Chemokine, (C-X-C motif) ligand 11 | 23.9996117 | 2.50345026 | 3.2610214 | 0.82069424 |
| GZMH | 2999 | Granzyme H (cathepsin G-like 2, protein h-CCPX) | 34.4991807 | 5.24225217 | 2.7183034 | 0.81805093 |
| CEACAM5 | 1048 | Carcinoembryonic antigen-related cell adhesion molecule 5 | 131.850262 | 30.9515392 | 2.0908174 | 0.81214063 |
| CXCL5 | 6374 | Chemokine (C-X-C motif) ligand 5 | 17.0501241 | 1.12837124 | 3.9174685 | 0.80810288 |
| PLAUR | 5329 | Plasminogen activator, urokinase receptor | 57.600887 | 12.0886809 | 2.2524342 | 0.80447282 |
| MIR4680 | 100616113 | MicroRNA 4680 | 14.2140901 | 0.31105917 | 5.5139889 | 0.80363476 |
| MMP1 | 4312 | Matrix metallopeptidase 1(intestinal collagenase) | 80.9107855 | 18.7769467 | 2.1073695 | 0.80093275 |

*log2ratio is the log_2_ of the ratio of FPKM values in HIV infected versus uninfected children; ^§^probability refers to the probability of that finding being a true finding in the NOIseq analysis

**Figure S2 Correlations of genes related to cell cycling/proliferation with villus height and permeability**

**Figure S3 Correlations of expression levels of genes related to immune activation with villus height, LR ratio, LPS or anti-DGP IgG**

**Figure S4** **Correlations of expression levels of solute carrier (SLC) genes with villus height or LR ratio**

**Figure S5** **Correlations of expression levels of genes related to mucus integrity and mucosal defence with villus height or LR ratio**

**Table S6 Correlations of expression levels of genes involved in xenobiotic metabolism with VH, LR or LPS**

**Figure S7** NetworkAnalyst^TM^ nodes and edges: villus height


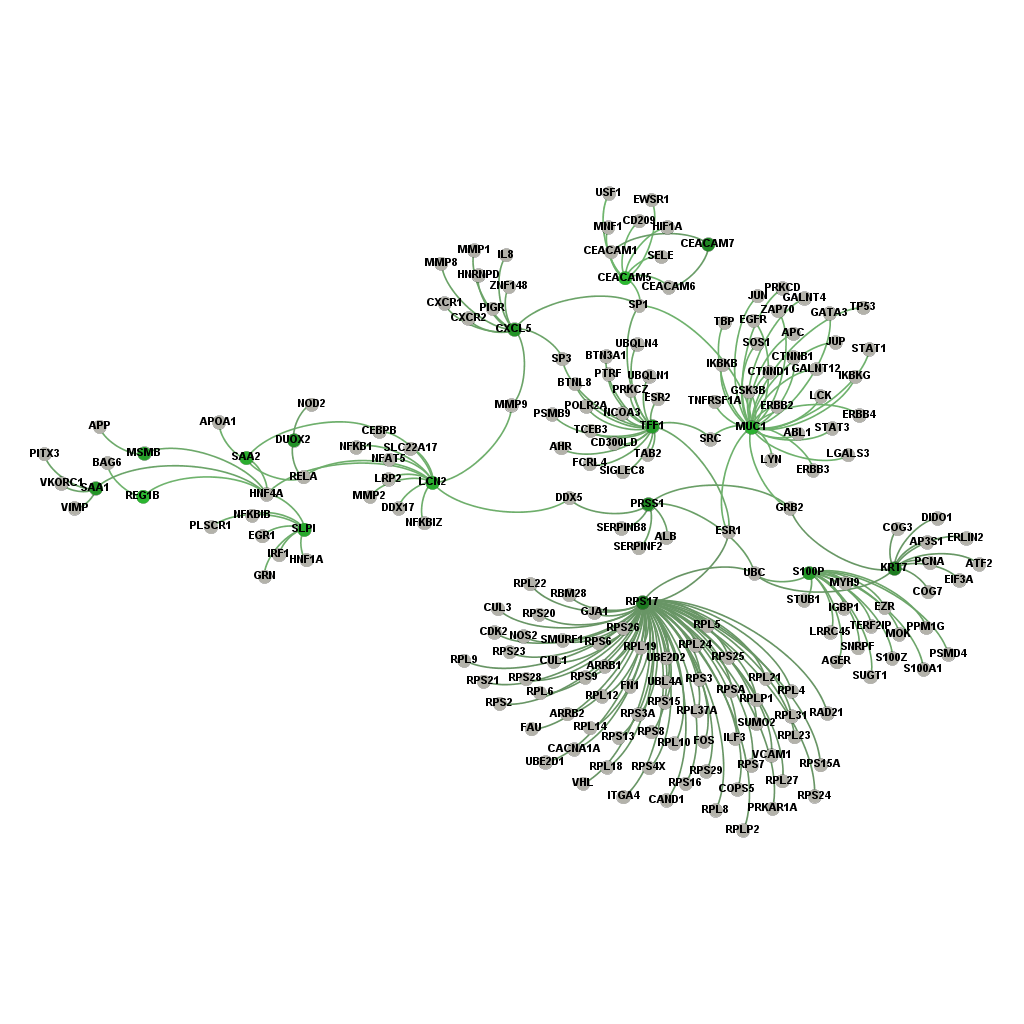


**Figure S8** NetworkAnalyst^TM^ nodes and edges: lactulose:rhamnose ratio


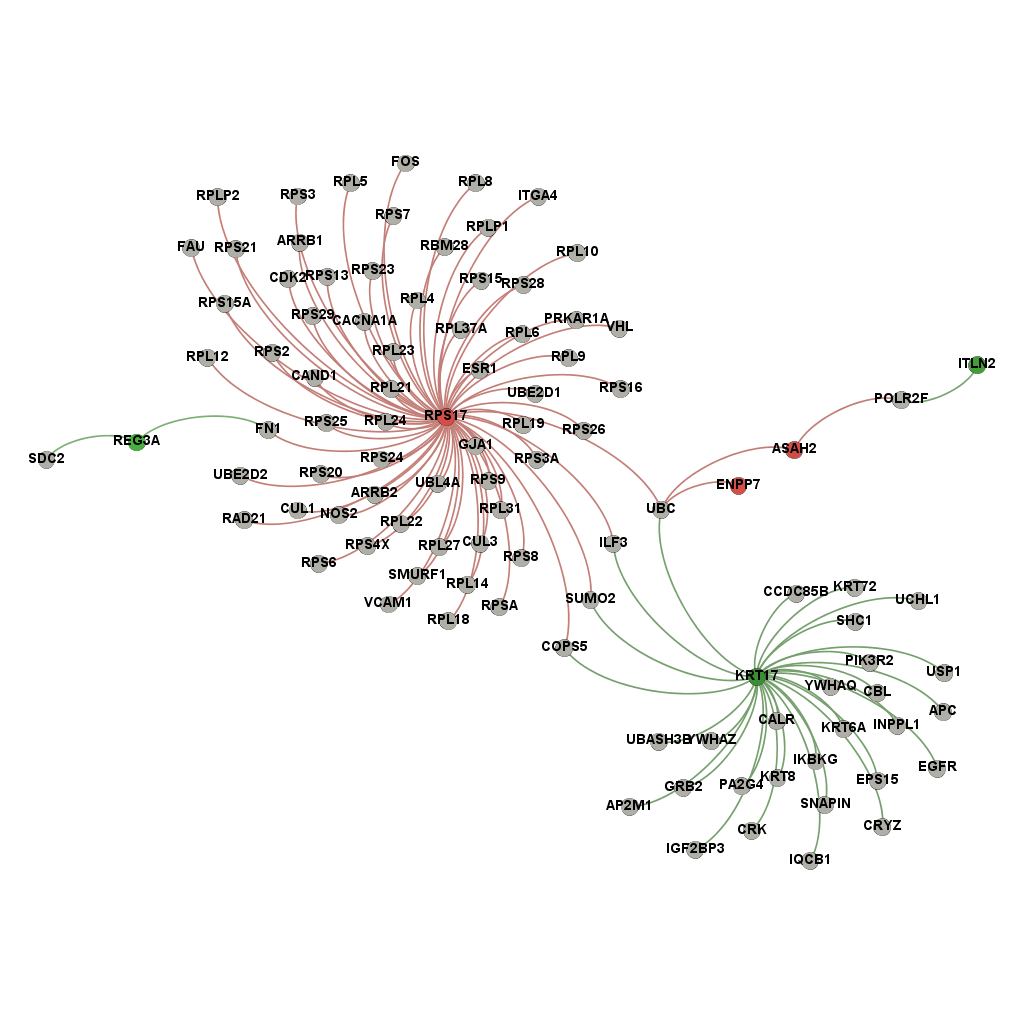


**Figure S9** NetworkAnalyst^TM^ nodes and edges: lipopolysaccharide


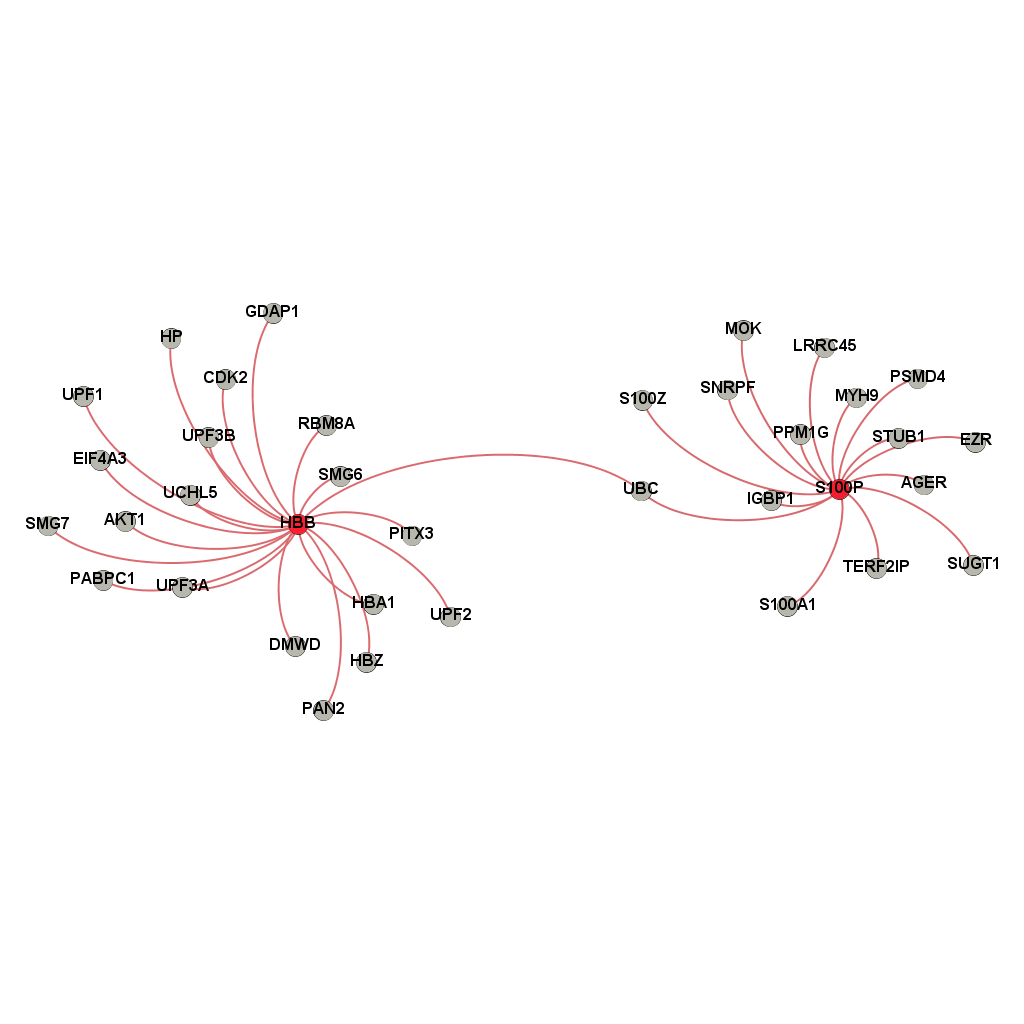


**Figure S10** NetworkAnalyst^TM^ nodes and edges: anti-DGP IgG


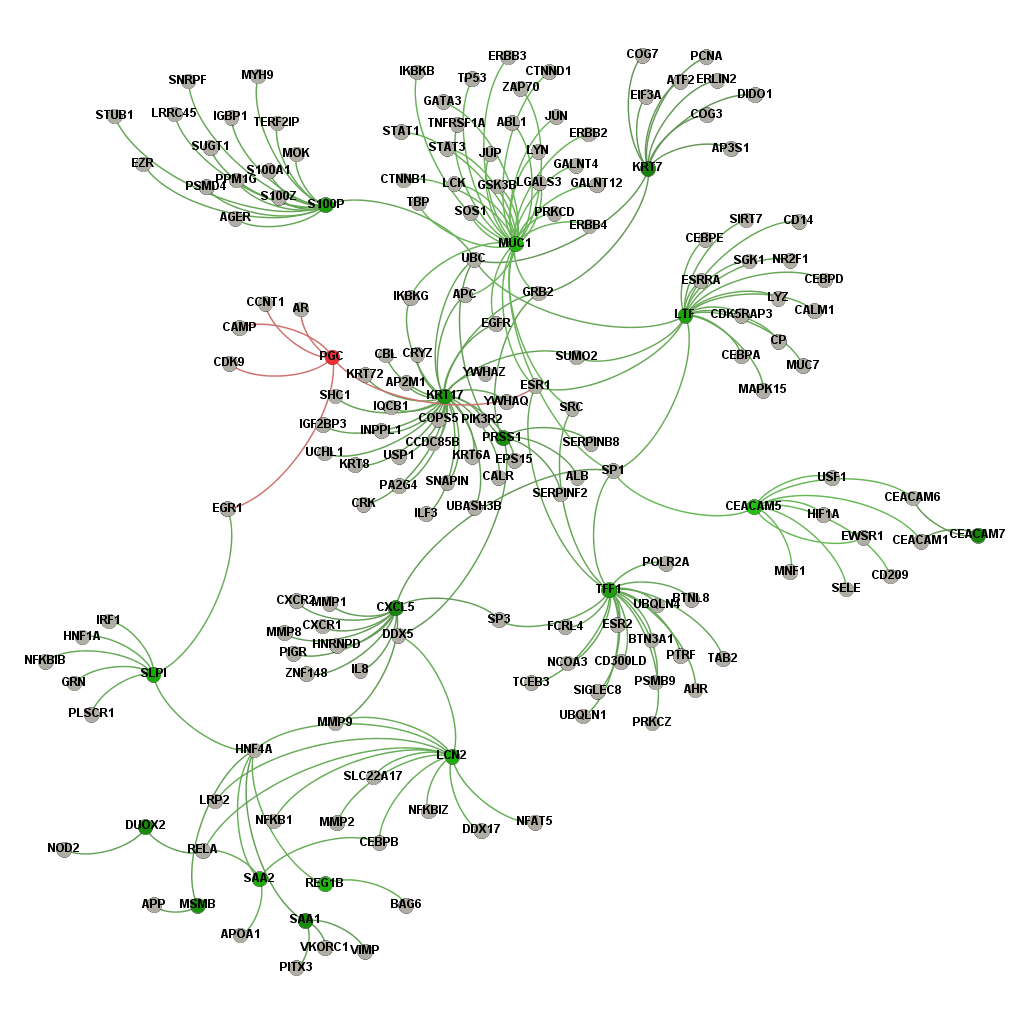


**Table S3** Summary of systematic review of original articles describing transcriptomic profiles in IBD in adults and children

| No | Disease group | Technology | Major findings | Ref |
| --- | --- | --- | --- | --- |
| Paediatric IBD | | | | |
| 1 | Crohn’s | RNA sequencing | Increased expression of DUOX2, DUOXA2, lipocalin 2, CEACAM 5 and 7, MUC1, SAA 1, 2 and 4, and CXCL5 genes | S#1 |
| 2 | IBD | Microarray | Increased expression of CXCL9, DUOX2, DUOXA2, SOD2 | S#2 |
| 3 | IBD | Microarray | Increased activity of IL-6 dependent pathways | S#3 |
| Adult IBD | | | | |
| 4 | Crohn’s | Microarray | Increased expression of FOXF2, MUC1, CDH11, HLA-A, HLA-B | S#4 |
| 5 | UC | Microarray | Increased expression of DUOX2 | S#5 |
| 6 | Crohn’s | RNA sequencing | Increased expression of CXC chemokines, S100A8, Reg1B in active disease | S#6 |
| 7 | Crohn’s | Microarray | IL23A and STAT3 upregulated in CD group. Solute transporters downregulated | S#7 |
| 8 | IBD | Microarray | Increased expression of REG1B, DEAF6, CXCLs | S#8 |

We conducted a systematic PubMed search for original research articles describing transcriptomic profiles, using the search terms “Crohn’s disease”, “Inflammatory Bowel Disease”, “transcriptome”, or “RNA sequencing”. We have only presented hits described as significantly associated with disease or with disease severity in the main text of the paper or in supplementary material; full datasets were not analysed *de novo*.

References for Table S3:

S#1 Haberman Y, et al. Pediatric Crohn disease patients exhibit specific ileal transcriptome and microbiome signature. J Clin Invest. 2014;124:3617–33

S#2 Fang K, Grisham MB, Kevil CG. Application of comparative transcriptional genomics to identify molecular targets for pediatric IBD. Front Immunol. 2015;6:165. doi: 10.3389/fimmu.2015.00165.

S#3 Carey R, Jurickova I, Ballard E, et al. Activation of an IL-6:STAT3-dependent transcriptome in pediatric-onset inflammatory bowel disease. Inflamm Bowel Dis. 2008;14(4):446-57.

S#4 Costello CM, Mah N, Häsler R, et al. Dissection of the inflammatory bowel disease transcriptome using genome-wide cDNA

microarrays. PLoS Med 2005;2:e199.

S#5 Mirza AH, Berthelsen CH, Seemann SE, et al. Transcriptomic landscape of lncRNAs in inflammatory bowel disease. Genome Med 2015;7:39.

S#6 Hong SN, Joung JG, Bae JS, et al. RNA-seq reveals transcriptomic differences in inflamed and noninflamed intestinal mucosa of Crohn’s disease patients compared with normal mucosa of healthy controls. Inflamm Bowel Dis 2017;23:1098-1108.

S#7 Noble CL, et al. Characterization of intestinal gene expression profiles in Crohn’s disease by genome-wide microarray analysis. Inflamm Bowel Dis. 2010;16(10):1717–1728. doi: 10.1002/ibd.21263

S#8 Clark PM, Dawany N, Dampier W, Byers SW, Pestell RG, Tozeren A. Bioinformatics analysis reveals transcriptome and microRNA signatures and drug repositioning targets for IBD and other autoimmune diseases. Inflamm Bowel Dis. 2012;18(12):2315–2333. doi: 10.1002/ibd.22958.
